# Comparison of the efficacy of nifedipine with ritodrine, nitroglycerine and magnesium sulfate for the management of preterm labor: a systematic review and meta-analysis

**DOI:** 10.1186/s12884-024-06497-w

**Published:** 2024-04-25

**Authors:** Maryam Zamani, Rasoul Alimi, Seyyed Mostafa Arabi, Maryam Moradi, Elham Azmoude

**Affiliations:** 1 Department of Midwifery, School of Nursing and Midwifery, Neyshabur University of Medical Sciences, Neyshabur, Iran; 2 Department of Epidemiology and Biostatistics, School of Health, Torbat Heydariyeh University of Medical Sciences, Torbat Heydariyeh, Iran; 3 Department of Nutrition, Neyshabur University of Medical Sciences, Neyshabur, Iran; 4 Global and Women’s Health, School of Public Health and Preventive Medicine, Faculty of Medicine, Nursing and Health Sciences, Monash University, Melbourne, Australia; 5 Neyshabur University of Medical Sciences, Bagcheh Ban town, Neyshabur, Iran

**Keywords:** Preterm birth, Tocolysis, Nifedipine, Nitroglycerin, Ritodrine, Nitroglycerin, Magnesium sulfate, Systematic review

## Abstract

**Background:**

Some studies have compared the efficacy of nifedipine with that of other tocolytic drugs in the treatment of preterm labor, but the reported results are conflicting.

**Objective:**

To compare the efficacy of nifedipine with that of ritodrine, nitroglycerine and magnesium sulfate for the management of preterm labor.

**Methods:**

In this systematic review and meta-analysis, PubMed/MEDLINE, Scopus, Clarivate Analytics Web of Science, and Google Scholar were searched until April 3,2024 using predefined keywords. Randomized controlled trials (RCTs) and clinical trials that compared the efficacy of nifedipine with that of ritodrine, nitroglycerine and magnesium sulfate for the management of preterm labor were included. Two authors independently reviewed the articles, assessed their quality and extracted the data. The quality of the included RCTs based on the Cochrane Risk of Bias Tool 1 for clinical trial studies. The risk difference (RD) with the associated 95% confidence interval (CI) was calculated. A forest plot diagram was used to show the comparative point estimates of nifedipine and other tocolytic drugs on the prevention of preterm labor and their associated 95% confidence intervals based on the duration of pregnancy prolongation. Study heterogeneity was evaluated by the I_2_ index, and publication bias was evaluated by Egger’s test.

**Results:**

Forty studies enrolling 4336 women were included. According to our meta-analysis, there was a significant difference in the prolongation of preterm labor within the first 48 h between the nifedipine group and the nitroglycerine group (RD, -0.04; 95% CI, -0.08 to -0.00; I^2^: 32.3%). Additionally, there were significant differences between nifedipine and ritodrine (RD, 0.11; 95% CI, 0.02 to 0.21; I^2^, 51.2%) for more than one week RD, 0.10; 95% CI, 0.03 to 0.19; I^2^, 33.2%) and for 34 weeks and more. The difference between nifedipine and magnesium sulfate was not significant in any of the four time points.

**Conclusions:**

Considering the superiority of nifedipine over ritodrine and nitroglycerine and its similar efficacy to magnesium sulfate for tocolysis, it seems that the side effects of these options determine the first drug line.

**Supplementary Information:**

The online version contains supplementary material available at 10.1186/s12884-024-06497-w.

## Introduction

Preterm birth is a major global health problem, is the leading cause of neonatal mortality and is one of the primary causes of long-term neonatal morbidity, which poses a substantial economic burden [1]. The prevalence of preterm labor ranges from 5 to 18% worldwide. During the last 20 years, its prevalence has increased, which seems to be related to several risk factors, including advanced maternal age and the use of assisted reproductive methods [2–4]. Strategies to decrease preterm birth include risk assessment, objective evaluation of threatened preterm labor, evidence-based interventions, and reduction of iatrogenic prematurity [5]. A number of therapeutic options for the prevention of preterm labor are being investigated [6].

Since uterine contractions are most commonly recognized as a precursor to preterm birth, the use of therapeutic interventions to stop uterine contractions was the first choice. Therefore, many drugs, including magnesium sulfate, calcium channel blockers, oxytocin antagonists, nonsteroidal anti-inflammatory drugs (NSAIDs), and beta-adrenergic receptor agonists, have been used to inhibit myometrial contractions [7]. After diagnosing preterm labor, clinicians can choose from a range of tocolytic drugs to delay labor. This allows time for the administration of corticosteroids to mature fetal lungs, which can reduce neonatal morbidity and mortality [8].

Knowledge of the safety and effectiveness of these medications is paramount [9]. A considerable number of studies have been carried out to compare nifedipine with other tocolytics (ritodrine, nitroglycerine and magnesium sulfate). Ritodrine hydrochloride is the only FDA-approved drug for preterm labor [10]; compared with nifedipine, it has not shown significant differences in efficacy in some studies [11–13], while other reports have shown that nifedipine is more effective than ritodrine as a tocolytic agent for preterm labor [14–16]. However, few studies have reported that ritodrine has a better effect on the cessation of uterine contractions [17, 18]. The results from the comparison of nifedipine with nitroglycerine [19, 20] or magnesium sulfate [21, 22] have also been controversial. In terms of side effects, although most studies have reported that nifedipine is safe [23, 24], the results for the other three tocolytics are conflicting.

Due to the high prevalence of preterm delivery and the conflicting results regarding the efficacy of medication, the present study aimed to compare the efficacy of nifedipine with ritodrine, nitroglycerine and magnesium sulfate for the management of preterm labor through a systematic review and meta-analysis.

## Methods

The methodology and reporting of this systematic review were based on the Preferred Reporting Items for Systematic Reviews and Meta-Analyses (PRISMA) statement and checklist [25]. The protocol for this review was registered at PROSPERO, the international prospective register of systematic reviews with registration (Supplementary file, S1).

### Search strategy

Systematic searches for published articles until April 3, 2024, were performed in PubMed/MEDLINE, Scopus, Clarivate Analytics Web of Science and Google Scholar for randomized controlled trials (RCTs) of tocolytic drug interventions for preterm labor. We used the combination of MeSH and keywords in our search strategy, including “Nifedipine” with “Nitroglycerin” OR “Ritodrine” OR Nitroglycerin OR Magnesium Sulfate AND prolongation of pregnancy AND Randomized controlled trials OR clinical trials (the complete search strategy is provided in the Supplementary File 2). Our search was conducted without time and language limitations, and to ensure a comprehensive literature search, we checked all related reference lists of the included articles and meta-analyses for additional studies.

### Inclusion criteria

Studies were selected if they (1) were RCTs or clinical trials; (2) were all pregnant women with preterm labor; (3) had a gestational age younger than 37 weeks; (4) had intact membranes; (3) compared the effect of tocolytic drugs (ritodrine, nitroglycerin or magnesium sulfate) with that of nifedipine on the prolongation of pregnancy; (4) provided sufficient information on the prolongation of pregnancy, including the frequency and percentage, reported at the end of the interventions in each group. The PICOS criteria (interventions, comparisons, outcomes, and study design) are provided in Supplementary File S3.

### Exclusion criteria

Studies were excluded if they (1) reported membrane rupture, (2) compared nifedipine with multiple tocolytic drugs, (3) did not report favourable outcomes in the intervention groups, or (4) were animal studies, observational studies, editorials, letters, or reviews.

### Screening and data extraction

Two independent review authors (M.Z. and R.A.) initially screened the retrieved titles and abstracts using a standardized Excel data abstraction form and then assessed the full texts of the selected studies for potential eligibility. EndNote software was used to export all the retrieved articles. Duplicates were removed, and multiple reports for the same trial were linked together as one study. Two review authors (M.Z. and R.A.) independently extracted data on the study design, baseline characteristics of the enrolled patients, risk of bias domains, and study outcomes, as well as the frequency and percentage of the main outcome (prolongation of pregnancy). In articles with insufficient data, we emailed the corresponding author twice at two-week intervals to obtain additional information.

Any disagreements were resolved through discussion and based on the opinion of a third researcher (E.A.). The following data were extracted from the study: first author’s name, year of publication, study location, intervention type, study design, characteristics of enrolled participants (numbers, mean age, gestational age), duration of prolongation of pregnancy (within 48 h, between 48 h and 7 days, more than 7 days, 34 weeks and more), and outcomes reported as frequency and percent.

### Risk of bias assessment

We evaluated the quality of the included RCTs based on the Cochrane Risk of Bias Tool 1 (ROB 1) for clinical trial studies [26]. Two independent authors (M.Z. and R.A.) assessed each article’s quality according to the seven items of ROB 1: (1) random sequence generation, (2) allocation concealment, (3) selective outcome reporting, (4) blinding of participants and personnel, (5) detection bias (blinding of evaluators), (6) incomplete outcome data, and (7) other probable sources of biases. For each study, a label of bias was defined (low risk, high risk, or unclear risk of bias) (Supplementary file, S4).

### Statistical analysis

Comparative point estimates (risk differences: RD) with 95% confidence intervals of the effects of tocolytic drugs versus nifedipine treatment on the prevention of preterm labor were extracted from all studies. Then, a forest plot diagram was used to show the results of each study as well as heterogeneity between studies. To estimate the pooled effect, assuming that the extracted articles were a random sample of the total population under study, a random effect model was used. Funnel plot diagrams and Egger’s regression asymmetry test were used to assess publication bias. Finally, sensitivity analysis was used to determine the impact of specific studies on the overall impact estimate. Statistical analysis was performed using Stata software, version 16.0 (Stata Corp. 2019. Stata Statistical Software: Release 16. College Station, TX: Stata Corp LLC), and a significance level of 0.05 was used.

## Results

### Study selection

**Fig. 1:**
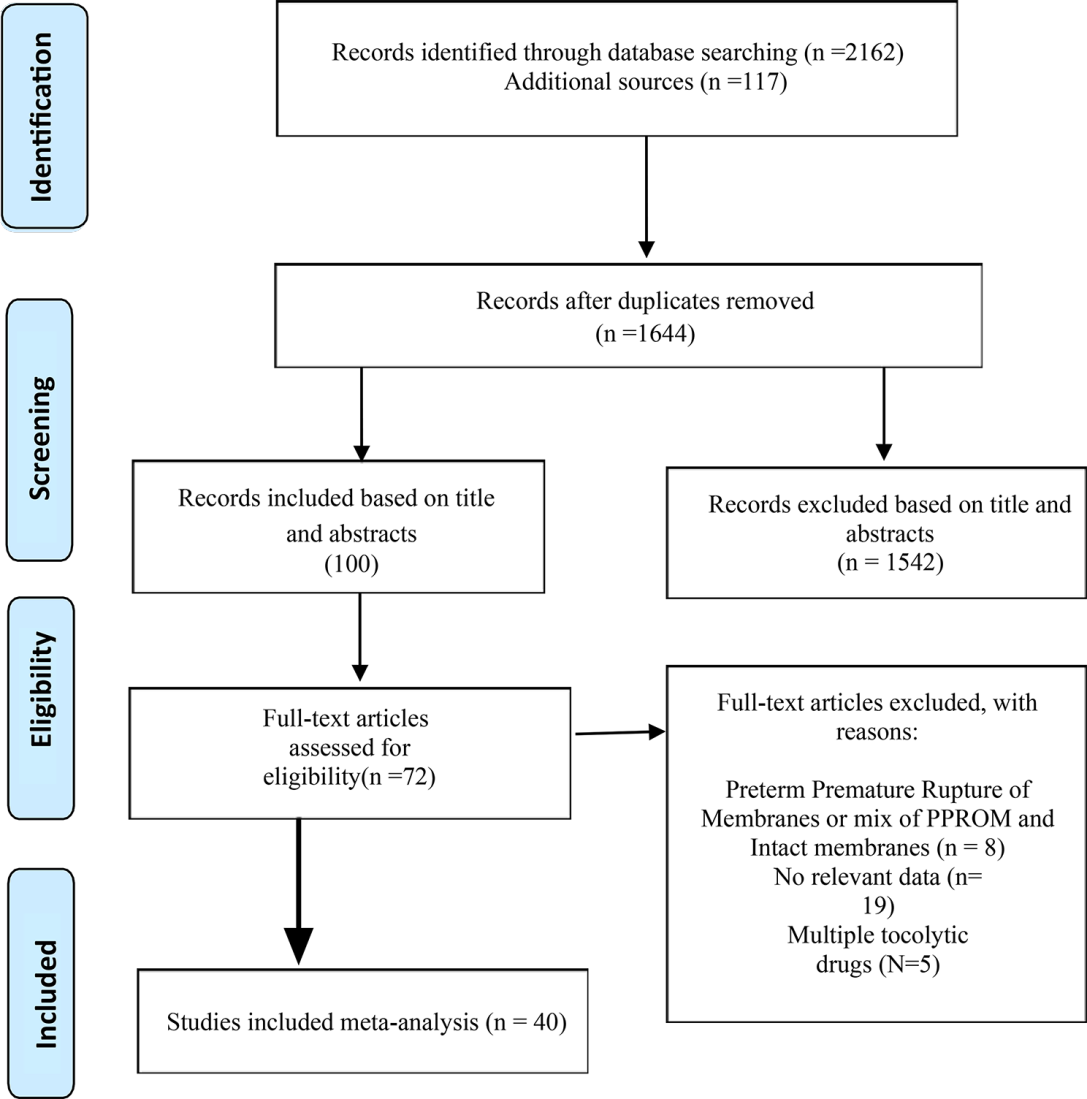
Flow diagram describing the study design process

After the initial search of all the databases, a total of 2162 articles were retrieved. Of those, 635 duplicate documents were excluded. The whole search process is shown in the PRISMA flow diagram in Fig. 1. After screening the remaining 100 titles and abstracts, we considered 72 articles for further evaluation of the full texts. We excluded 32 studies because 8 studies included preterm labor with membrane rupture or because they did not separate intact from ruptured membranes and reported mixed data; 19 studies did not include relevant data, and 5 studies used multiple tocolytic drugs. Finally, 40 favourable trials were included in the pooled analysis [14, 16, 17, 19, 22, 27–61].

### Study characteristics

The features of the 40 trials included in the present study are indicated in Table 1 [14, 16, 17, 19, 22, 27–61]. The trials were published between 1991 and 2023. The total number of participants ranged between 42 and 200, with a total sample size of 4336 participants. The sample sizes of the studies that compared nifedipine and nitroglycerin were 1791 participants, 902 for nifedipine and ritodrine, and 1643 for nifedipine and magnesium sulfate. The minimum age of the participants was 16 years, the maximum age was 42 years, and the gestational age ranged between 20 and 37 weeks.

**Table 1:** Characteristics of the included trials comparing the efficacy of nifedipine with that of ritodrine, nitroglycerine and magnesium sulfate for the management of preterm labor

Study author’s	Publication year	Country	Intervention	Sample size	Age Mean or range			Gestational age Mean or range				Prolongation of labor More than 48 h percent (%)		Reference
					N^*^	NG^*^/R^*^/M^*^		N	NG/R/M			N	NG/R/M	
Akhtar	2020	Pakistan	N- NG	126	28.7	29.9		32.1	32.3			-	-	[49]
Badshah	2019	Pakistan	N- NG	154	29.5			Not reported				54.5	59.7	[50]
Balasubramani	2017	India	N- NG	100	23.9	24.0		28–36				22	14	[24]
Dhawle	2013	India	N- NG	84	26.2	25.8		31.3	31.1			-	-	[28]
Iftikhar	2017	Pakistan	N- NG	72	26.5			26–35						[33]
Jamil	2020	Pakistan	N- NG	100	26.0	30.4		31.2	31.4			-	-	[16]
Kashanian	2014	Iran	N- NG	120	26.0	24.0		31.4	31.5			68.3	86.7	[35]
Kaur	2021	India	N- NG	100	20–29			28–34				40	60	[36]
Khan	2019	Pakistan	N- NG	200	20–40			Not reported				58	54	[44]
Sharma	2019	India	N- NG	100	24.4	24.1		32.8			33			[40]
Zulfiqar	2016	Pakistan	N- NG	60	30.5	28.1		30.5	29.1			23.3	33.3	[43]
Padmini	2015	India	N- NG	195	Without restriction			28–36				-	-	[39]
Vinodhini	2019	India	N- NG	80	28–37			28–36				15	5	[41]
Yasmin	2016	Pakistan	N- NG	50	Not reported			28–34				8	16	[42]
Goyal	2023	India	N- NG	130	24.4		24.9	28–37				61	65	[58]
Kalburgi	2023	India	N- NG	120	24.2		24.2	28–36				70	61.67	[60]
Al-Qattan	2000	Kuwait	N- R	60	Without restriction			29.4	29			-	-	[13]
Bankatlal	2011	India	N- R	120	16–25			28–36				90	68.3	[34]
Ceyhan	2007	Turkey	N- R	135	28.9	28.6		33.2	34.0			80.8	62.9	[27]
Garcıa-Velasco	1998	Spain	N- R	52	31.2	29.4		31.2	29.4			88.4	92.3	[30]
Gurjar	2017	India	N- R	100	16–40			28–36				80	68	[32]
Kupfermi	1993	Israel	N- R	60	28.4	28.6		30.1	30.2			83	77	[47]
Maitra	2007	India	N- R	70	Not reported			Under 34				2.9	17.1	[11]
Papatsoni	1997	Netherlands	N- R	185	28.7	29.8		28.8	29.5			37	19.1	[53]
Bracero	1991	USA	N- R	42	24.0	26.0		20–36				-	-	[26]
Cararach	2005	Spain	N- R	78	26.9	26.2		32.2	32.1			-	-	[14]
DikshaAmbedkar	2022	India	N- M	200	Without restriction			24–37				9	5	[54]
Glock	1993	USA	N- M	80	21.4	20.5		30.2	30.4			92	93	[31]
Kara	2009	Turkey	N- M	76	26	24.7		31.1	30.7			84.2	87.5	[52]
Khan	2021	Pakistan	N- M	180	32	32		35			35	-	-	[45]
Klauser	2013	USA	N- M	199	22.2	23.9		28.6			28.6	-	-	[46]
Klauser	2015	USA	N- M	60	23.6	22.7		30.1			30.8	59.3	60.6	[19]
Nikbakht	2014	Iran	N- M	100	Without restriction			24–37				8	4	[37]
Niroomanesh	2001	Iran	N- M	46	25.5	23.9		33.3			33.9	91.3	78.2	[38]
Faraji	2013	Iran	N- M	100	24.4	23.8		32.6			31.6	72	48	[29]
Subhashini	2013	India	N- M	200	Without restriction			28–34				-	-	[48]
Vinodhini	2019	India	N- M	80	18–40			24–28				15	2	[41]
Alavi	2015	Iran	N- M	64	Not reported			25–34				90.6	90.6	[51]
Bhat	2023	India	N- M	80	23		25	28–37				85	80	[59]
Saleem	2023	Pakistan	N- M	178	25.9		26.4	33.1		32.5		-	-	[61]

N: Nifedipine NG: Nitroglycerin M: Magnesium sulfate R: Ritodrine

### Meta-analysis

**Fig. 2:**
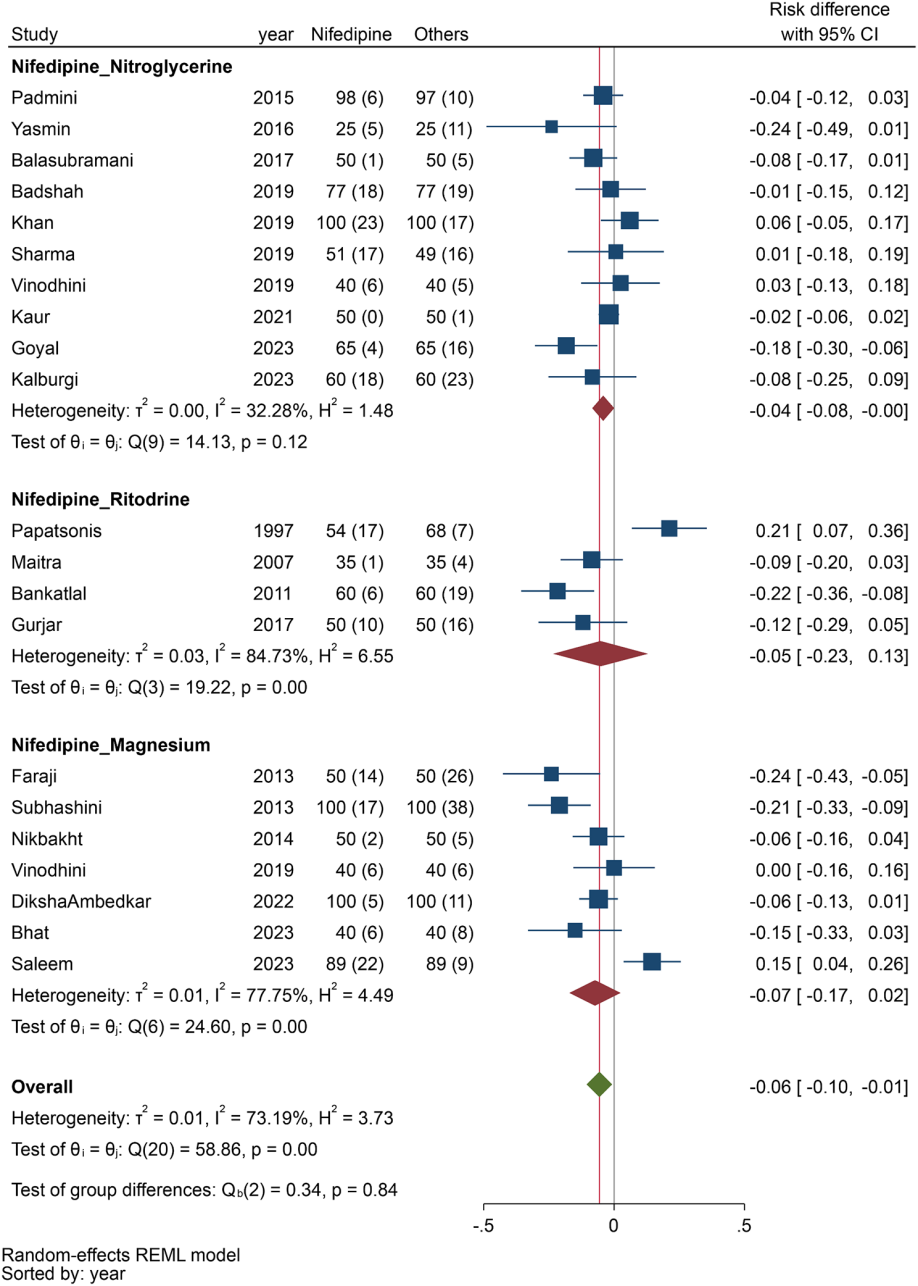
Comparing the efficacy of nifedipine with other tocolytic drugs in prolongation of pregnancy within 48 h

According to our meta-analysis (Fig. 2), which examined the status of preterm labor within the first 48 h, which included 21 studies, preterm labor was significantly lower in the nifedipine group than in the other tocolytic drug group (RD, -0.06; 95% CI, -0.10 to -0.01; I^2^: 73.2%). Subgroup analysis revealed that this difference was due to a significant difference between nifedipine and nitroglycerine (RD, -0.04; 95% CI, -0.08 to -0.00; I^2^: 32.3%).

**Fig. 3:**
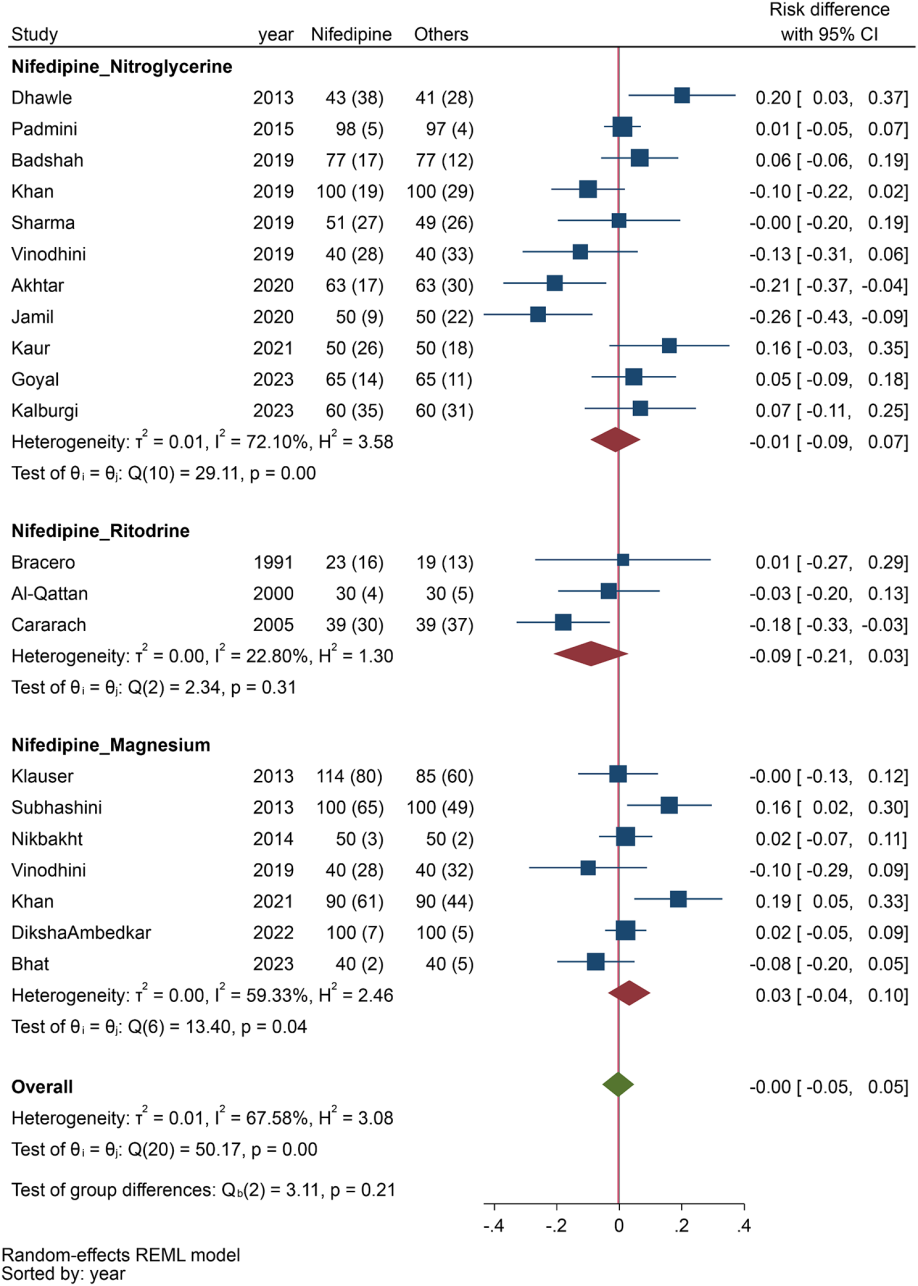
Comparing the efficacy of nifedipine with other tocolytic drugs in prolongation of pregnancy labor within 48 h to a week

The pooled analysis (Fig. 3), which examined the status of preterm labor within 48 h to a week, included nineteen studies, analysis showed that the rate of prolonged preterm delivery was similar between nifedipine and other tocolytic drugs (RD, -0.00, 95% CI, -0.05 to 0.05; I^2^: 67.6%).

**Fig. 4:**
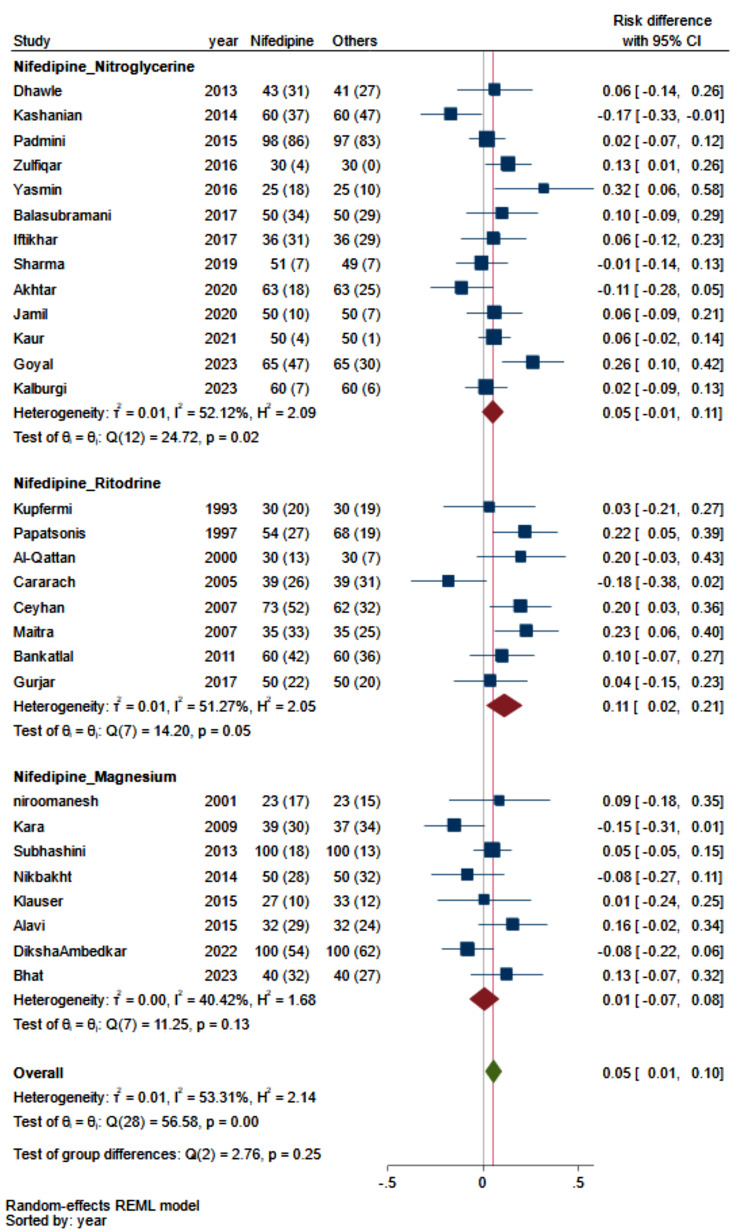
Comparing the efficacy of nifedipine with other tocolytic drugs in prolongation of pregnancy labor for more than 1 week

According to our meta-analysis (Fig. 4), which examined the status of preterm labor for more than one week and included twenty-nine studies, the prolongation of preterm delivery was 5% greater for patients receiving nifedipine than for those receiving other tocolytic drugs (RD, 0.05; 95% CI, 0.01 to 0.10; I^2^: 53.3%). Subgroup analysis revealed that this difference was due to differences between nifedipine and ritodrine (RD: 0.11; 95% CI, 0.02 to 0.21; I^2^:51.3%).

**Fig. 5:**
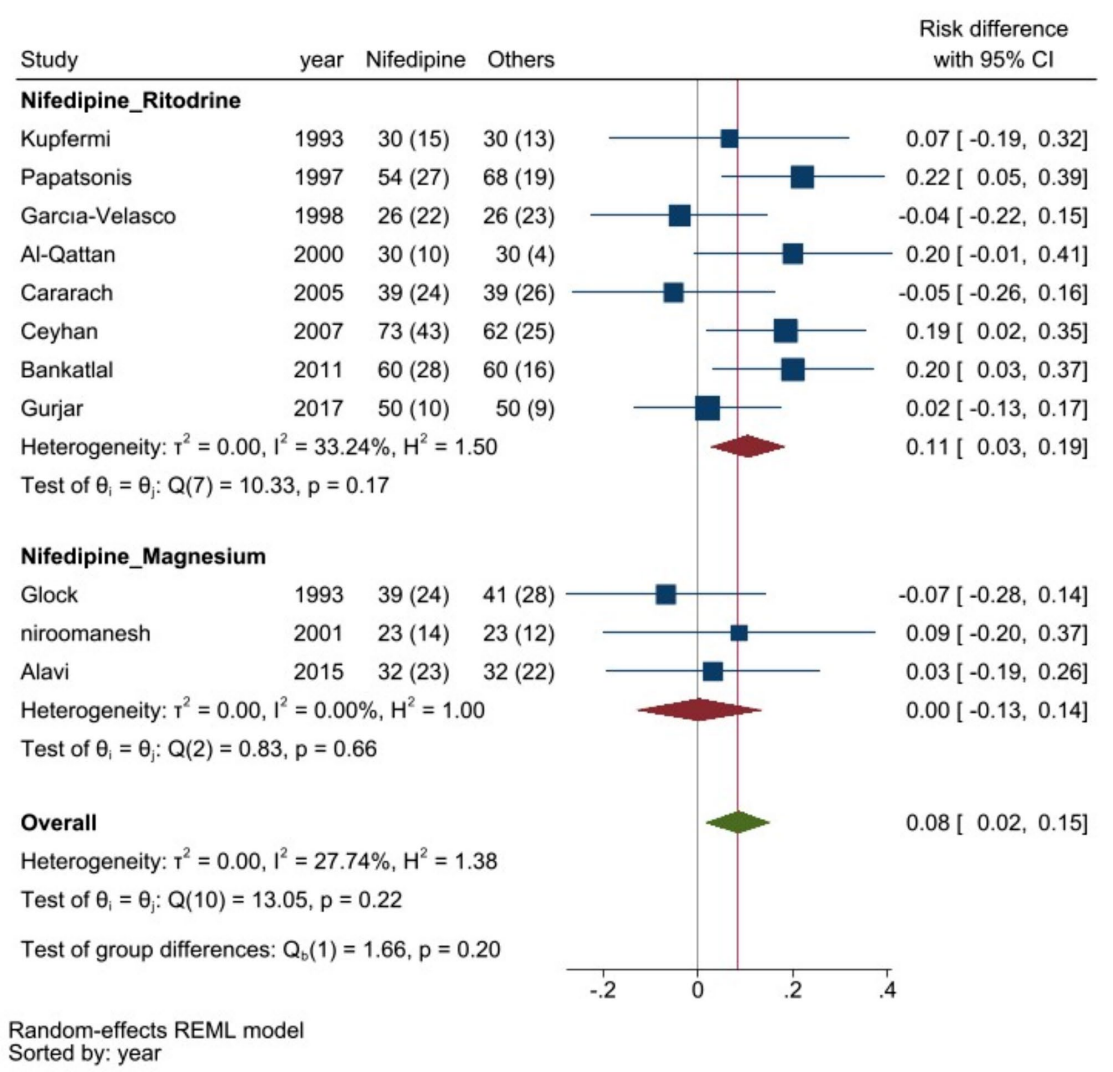
Comparing the efficacy of nifedipine with other tocolytic drugs in prolongation of pregnancy labor for 34 weeks and more

According to the pooled analysis (Fig. 5), which examined the status of preterm labor for 34 weeks or more, which included eleven studies, subgroup analysis revealed that the rate of prolonged preterm delivery was significantly greater (11%) for nifedipine than for ritodrine (RD: 0.11; 95% CI, 0.03 to 0.19; I^2^:33.2%).

Subgroup analysis based on low risk of bias studies revealed that the difference in the rate of prolonged preterm delivery between patients receiving nifedipine and patients receiving magnesium sulfate or nitroglycerine was not statistically significant across all four time points. However, the rate of prolonged preterm delivery was approximately 14% greater for nifedipine than for ritodrine in two time periods, “more than 1 week” and “34 weeks and more”, which was a statistically significant difference (supplementary file, S9-S12).

### Publication bias and influence tests

In all the meta-analyses, the funnel plots showed no publication bias, which was also confirmed by Egger’s test (*p* > 0.05) (Supplementary File, S5-S8). The sensitivity tests by leave-one-out meta-analysis indicated that all single-study omitted estimates lay within the 95% CI of the respective overall effect. This suggested that the pooled effect was not substantially influenced by any single study.

## Discussion

This study examined the efficacy of nifedipine in comparison with ritodrine, nitroglycerine and magnesium sulfate for the management of preterm labor through a systematic review and meta-analysis. Although tocolytics were introduced as the best way to suppress preterm labor many years ago, obstetricians are still not sure which tocolytic agent is the best available option [62].

The results of the present study showed that tocolysis with nifedipine is associated with lower preterm birth than that associated with other drugs within the first 48 h after the start of contractions. This result was due to a significant difference between nifedipine and nitroglycerine. As mentioned, tocolytic agents should be able to postpone pregnancy for at least 48 h to provide adequate time to administer antenatal corticosteroids, which would help boost the maturity of the fetal lung and prevent respiratory distress syndrome in the newborn [63, 64].

In contrast with the findings of this study, Conde-Agudelo et al., in a published meta-analysis in 2013, reported that there were no significant differences between oral nifedipine and transdermal GTN in terms of pregnancy prolongation and delivery within 48 h of treatment [65]. In a systematic review that included several studies in the present review, Flenady et al. (2014) reported that in comparison with other tocolytics (including betamimetics, nitroglycerin, nonsteroidal anti-inflammatory drugs (NSAIDs), magnesium sulfate and oxytocin receptor antagonists (ORAs)), calcium channel blockers (mainly nifedipine) did not result in a significant reduction in preterm birth. However, since the findings of the present meta-analysis include more recent studies, the resulting evidence seems to be more reliable [66].

On the other hand, the analysis of studies on the efficacy of tocolytics in delaying childbirth for 48 h to one week revealed that nifedipine is not more effective than other tocolytics. Similarly, a Cochrane review on the efficacy of nifedipine compared to other tocolytics in inhibiting preterm birth in 2014 showed that there was no significant evidence that nitric oxide donors were more successful than nifedipine in prolonging pregnancy beyond 48 h [67].

However, in contrast, Conde-Agudelo et al. (2011), in a published systematic review and meta-analysis of twenty-six trials and 2179 women, reported that nifedipine was associated with a significant reduction in the risk of delivery within 7 days of initiation of treatment and before 34 weeks gestation compared with β_2_-adrenergic receptor agonists. Similarly, there was also no difference between nifedipine and magnesium sulfate in terms of tocolysis efficacy in this study [68].

Conversely, the results of two previous systematic reviews by Oei and Tsatsaris in 1999 and 2001 showed that nifedipine was more effective at delaying labor beyond 48 h than ritodrine, which reflects the changes in evidence from recent studies [69, 70].

In contrast, in the included studies that examined the status of preterm labor for more than a week, the prolongation of pregnancy rate was 11% greater in the nifedipine group than in the ritodrine group. On the other hand, the results showed that nifedipine was 11% more effective at reducing the risk of delivery before 34 weeks than ritodrine, which was a statistically significant difference. This suggests that the long-term efficacy of nifedipine on preventing preterm birth is greater than that of ritodrine.

Similarly, a Cochrane review of 12 randomized controlled trials from 2003 involving a total of 1029 women showed that nifedipine is more effective than ritodrine in prolonging pregnancy beyond 7 days and is much less likely to cause maternal side effects [71].

Generally, although some studies have recommended nifedipine as the first-line tocolytic therapy, our study revealed no difference among nifedipine, nitroglycerin and ritodrine in delaying preterm labor for the first week. However, it was more effective than magnesium sulfate, only delaying labor for 48 h. However, the difference in the effect of these two drugs did not reach a significant level after analysing only the studies with a low risk of bias. In addition, the long-term tocolytic effects of nifedipine for postponing preterm birth for more than one week were not different from those of nitroglycerine or magnesium sulfate. A meta-analysis of studies with a low risk of bias confirmed the same findings and only showed the superiority of nifedipine over ritodrine in delaying birth for more than one week or more than 34 weeks. Therefore, the maternal and neonatal side effects of these three drugs can determine the first choice for tocolytic therapy.

In the present study, it was not possible to analyse the side effects of these drugs due to the extent of the mentioned side effects and the lack of uniformity in how they are reported. In this regard, the results of many studies have shown that the risk of pulmonary edema, a serious maternal side effect, is greater for beta-mimetic drugs and magnesium sulfate than for nifedipine. The next issue is that many of the neonatal complications mentioned in the studies can be due to the premature nature of the newborn and not the complications of the drugs. However, according to some studies, the risk of dangerous neonatal complications such as necrotizing enterocolitis (NEC), respiratory distress syndrome (RDS) and intraventricular hemorrhage (IVH) is lower with nifedipine [68, 72].

### Strengths and limitations

The present study has several strengths. An extensive literature search was conducted using different databases, and as a result, many existing studies were included in this analysis. In addition, the use of subgroup analysis to reduce the heterogeneity of findings was another strength of this study. In addition, the quality of the articles was assessed, and the analysis was performed on all the studies as well as on the studies with a low risk of bias. In addition, the results of the relevant tests were not biased.

In addition, some limitations in the present study should be considered. First, the heterogeneity was high, so the subgroup analysis was performed based on the duration of delivery delay and the risk of bias in the studies. Second, the criteria used for preterm labor differed based on uterine contractions and cervical changes. The dosage of drugs and the duration of treatment were also different among the included studies. Finally, since the aim of this study was to review all the evidence, the limitation of maternal age was not applied to the selection of studies.

Further studies are recommended to compare the effectiveness of different doses of tocolytic drugs with fewer side effects on maternal and foetal outcomes. In addition, higher-quality, blinded trial studies that demonstrate maternal safety and offspring short- and long-term outcomes of tocolytics are also recommended.

## Conclusions

In general, the results of the present study showed that nifedipine is more effective than magnesium sulfate in postponing preterm labor within 48 h. However, this drug was equally effective in postponing preterm birth compared to other tocolytics, including nitroglycerin and ritodrine, during this period. On the other hand, the efficacy of nifedipine was not different from other tocolytics for delaying labor between 48 h and one week. However, based on the findings of this meta-analysis, the effectiveness of nifedipine for long-term prevention of preterm birth for more than one week and more than 34 weeks was greater than ritodrine but equal to magnesium sulfate and nitroglycerin.

### Electronic supplementary material

Below is the link to the electronic supplementary material.

Supplementary Material 1

## Data Availability

The supplementary material is available. The dataset used in this study is available from the corresponding author upon request.
