# Supplementary material for: Comparison of the efficacy of nifedipine with ritodrine, nitroglycerine and magnesium sulfate for the management of preterm labor: a systematic review and meta-analysis
Source: BMC Pregnancy Childbirth. 2024 Apr 25;24:318. doi: 10.1186/s12884-024-06497-w (PMC11044545; doi:10.1186/s12884-024-06497-w)

**Supplementary file (S1-S12)**

**S1:** PROSPERO registration

**We registered three study in PROSPERO separately but for publication of results we decided to combine the results of these studies together in one article. The titles and PROSPERO ID of each study is reported here.**

1. Comparison of the effectiveness of nifedipine and transdermal nitroglycerin in treatment of preterm labor: a systematic review and meta-analysis PROSPERO ID: CRD42021230008
2. The comparison of efficacy of nifedipine and ritodrine on preterm labor: systematic review and meta-analysis PROSPERO ID: CRD42021290766
3. The comparison of efficacy of nifedipine and magnesium sulfate on preterm labor: systematic review and meta-analysis PROSPERO ID: CRD42021290768

**S2:** Example search strategy

**PubMed**

**Search: ((((((((birth, preterm[MeSH Terms] ) OR (births, preterm[MeSH Terms])) OR (labor, preterm[MeSH Terms])) OR (preterm birth[MeSH Terms])) OR (preterm births[MeSH Terms])) OR (preterm labor[MeSH Terms])) OR ((preterm delivery[Title/Abstract]) OR (preterm[Title/Abstract]))) AND (((nifedipine[Title/Abstract]) OR (nifedipine[MeSH Terms])) OR (((uterine contraction[MeSH Terms]) OR (uterine contractions[MeSH Terms])) OR (tocolysis[MeSH Terms])))) AND (((((Magnesium Sulfate[MeSH Terms]) OR (Magnesium Sulfate[Title/Abstract])) OR ((ritodrine[MeSH Terms]) OR (ritodrine[Title/Abstract]))) OR ((nitroglycerin[MeSH Terms]) OR (nitroglycerin[Title/Abstract]))) OR (((uterine contraction[MeSH Terms]) OR (uterine contractions[MeSH Terms])) OR (tocolysis[MeSH Terms])))**

**S3:** PICOS criteria for inclusion of studies.

| PICOS criteria | Definitions |
| --- | --- |
| Participants | All Pregnant women with preterm labor |
| Intervention | Nitroglycerin OR Ritodrine OR Magnesium Sulfate |
| Comparator | Control drug (Nifedipine) |
| Outcomes | Prolongation of pregnancy |
| Study design | RCTs (parallel, and prospective) |

**S4:** Risk of bias assessment (ROB1).

| **Studies** | **Random sequence generation** | **Allocation concealment** | **Selective reporting** | **Other sources of bias** | **Blinding (participants and personnel)** | **Blinding (outcome assessment)** | **Incomplete outcome data** | **General risk of bias** |
| --- | --- | --- | --- | --- | --- | --- | --- | --- |
| Akhtar, 2020 | L | U | H | U | U | U | L | Unclear |
| Badshah, 2019 | L | L | L | U | U | U | L | LOW |
| Balasubramani, 2017 | L | H | U | U | U | U | L | Unclear |
| Dhawle, 2013 | L | L | H | L | U | U | L | LOW |
| Iftikhar, 2017 | L | L | H | L | U | U | L | LOW |
| Jamil, 2020 | L | L | H | L | U | U | L | LOW |
| Kashanian, 2014 | L | L | H | L | H | L | L | LOW |
| Kaur, 2021 | L | L | L | L | H | L | L | HIGH |
| Khan, 2019 | L | L | H | L | U | U | L | LOW |
| Sharma, 2019 | L | L | L | L | L | L | L | LOW |
| Zulfiqar, 2016 | H | H | L | L | U | U | L | HIGH |
| Padmini, 2015 | L | L | L | L | U | U | L | LOW |
| Vinodhini, 2019 | L | L | H | L | U | U | L | LOW |
| Yasmin, 2016 | L | U | H | L | U | U | L | Unclear |
| Goyal, 2023 | L | H | H | U | U | U | L | HIGH |
| Kalburgi, 2023 | L | H | H | U | U | U | L | HIGH |
| Al-Qattan, 2000 | L | L | L | L | U | U | L | LOW |
| Bankatlal, 2011 | L | L | L | L | U | U | L | LOW |
| Ceyhan, 2007 | H | H | H | L | H | H | L | HIGH |
| Garcıa-Velasco, 1998 | L | L | L | L | U | U | L | LOW |
| Gurjar, 2017 | H | H | H | L | U | U | L | HIGH |
| Kupfermi, 1993 | L | L | H | L | U | U | L | LOW |
| Maitra, 2007 | L | L | L | L | H | H | L | HIGH |
| Papatsoni, 1997 | L | L | L | L | U | U | L | LOW |
| Bracero, 1991 | L | L | L | L | U | U | L | LOW |
| Cararach, 2005 | L | L | L | L | H | H | L | HIGH |
| DikshaAmbedkar, 2022 | L | L | H | L | U | U | L | LOW |
| Glock, 1993 | L | L | L | L | U | U | L | LOW |
| Kara, 2009 | L | L | H | L | U | U | L | LOW |
| Khan, 2021 | L | U | H | L | U | U | L | Unclear |
| Klauser, 2013 | L | L | L | L | H | H | L | HIGH |
| Klauser, 2015 | L | L | L | L | H | H | L | HIGH |
| Nikbakht, 2014 | L | L | L | L | U | U | L | LOW |
| Niroomanesh, 2001 | U | U | H | L | U | U | L | Unclear |
| Faraji, 2013 | L | U | H | L | U | U | L | Unclear |
| Subhashini, 2013 | L | L | L | L | U | U | L | LOW |
| Vinodhini, 2019 | L | L | L | L | U | U | L | LOW |
| Alavi, 2015 | L | L | H | L | L | H | L | LOW |
| Bhat, 2023 | L | H | H | L | U | U | L | HIGH |
| Saleem, 2023 | L | H | H | L | U | U | L | HIGH |

**S5:** The funnel plot related to studies that compared the efficacy of nifedipine with other tocolytic drugs in prolongation of pregnancy within 48 hours

**S6** The funnel plot related to studies that compared the efficacy of nifedipine with other tocolytic drugs in prolongation of pregnancy within 48 hours to 1 week

**S7:** The funnel plot related to studies that compared the efficacy of nifedipine with other tocolytic drugs in prolongation of pregnancy to more than 1 week

**S8:** The funnel plot related to studies that compared the efficacy of nifedipine with other tocolytic drugs in prolongation of pregnancy for 34 weeks and more

**S9:** Comparing the efficacy of nifedipine with other tocolytic drugs in prolongation of pregnancy within 48 hours in low risk of bias studies


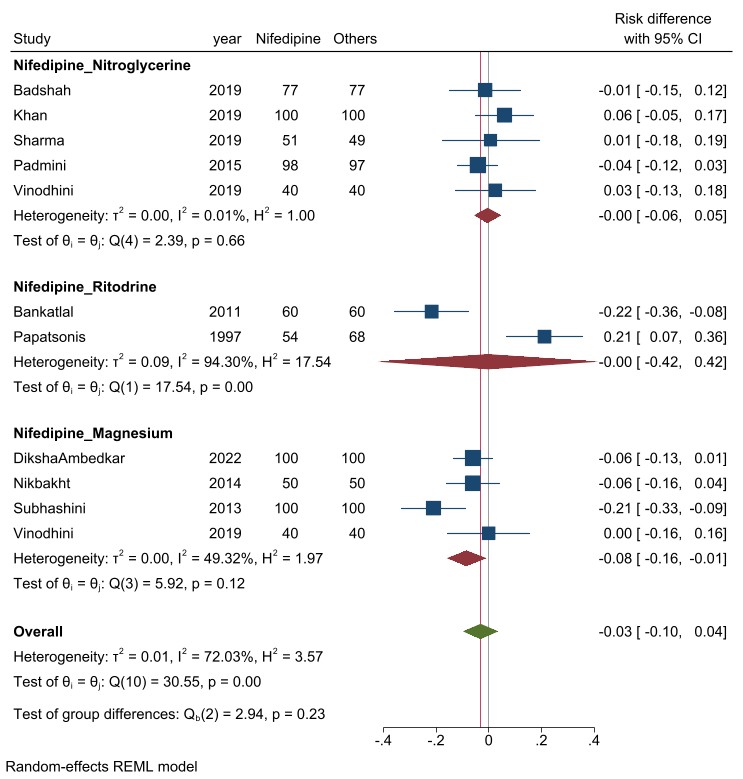


**S10**: Comparing the efficacy of nifedipine with other tocolytic drugs in prolongation of pregnancy within 48 hours to 1 week in low risk of bias studies


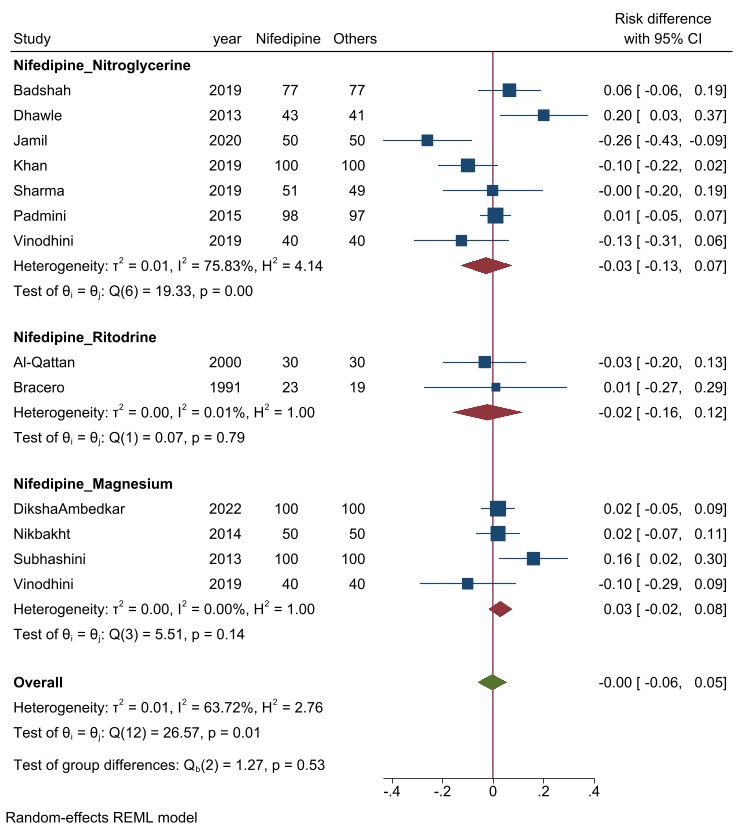


**S11:** Comparing the efficacy of nifedipine with other tocolytic drugs in prolongation of pregnancy for more than 1 week in low risk of bias studies


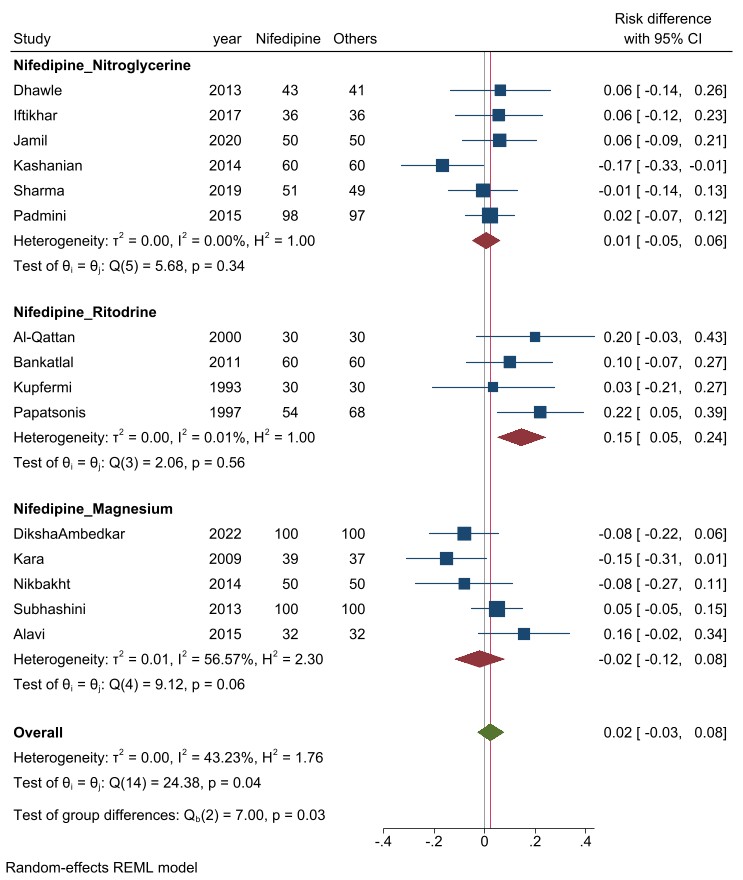


**S12:** Comparing the efficacy of nifedipine with other tocolytic drugs in prolongation of pregnancy for 34 weeks and more in low risk of bias studies


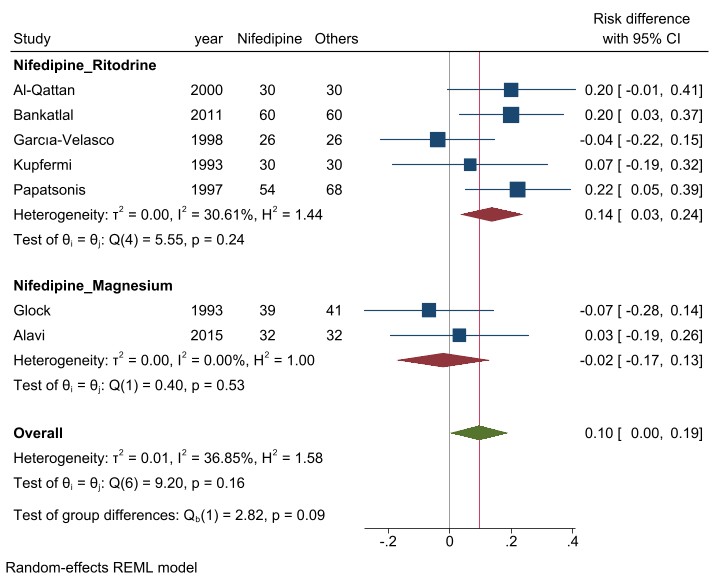

Supplement: Supplementary file 1 — Supplementary Material 1 [file 12884_2024_6497_MOESM1_ESM.docx]
